# An Economic Analysis of Mumps Vaccination in Fiji: Static Model Simulation of Routine Measles–Mumps–Rubella (MMR) Vaccination Instead of Current Measles–Rubella (MR) Vaccination

**DOI:** 10.3390/ijerph19031861

**Published:** 2022-02-07

**Authors:** Chunghyeon Oh, Eric Rafai, Yinseo Cho, Damin Jun, Seungman Cha

**Affiliations:** 1 Department of Surgery, CWM Hospital, 1 Waimanu Road, Suva, Fiji; och7512@gmail.com; 2 Fiji Office, Korea International Cooperation Agency, 91 Gordon St., Suva, Fiji; 3 Department for Research, Innovation, Data Analysis Management & Information Technology, Ministry of Health & Medical Services, 88 Amy Street, Suva, Fiji; eric.rafai@govnet.gov.fj; 4 West Africa Division, Korea International Cooperation Agency, Seongnam-si 13449, Korea; send2ulv@gmail.com; 5 Performance Management Team, Korea International Cooperation Agency, Seongnam-si 13449, Korea; jdm@koica.go.kr; 6 Department of Global Development and Entrepreneurship, Graduate School of Global Development and Entrepreneurship, Handong Global University, Pohang 37554, Korea; 7 Department of Disease Control, London School of Hygiene & Tropical Medicine, London WC1E 7HT, UK

**Keywords:** mumps vaccination, Fiji, cost benefit analysis, static model

## Abstract

Mumps remains endemic in Fiji, with 7802 cases reported between 2016 and 2018. The introduction of mumps vaccination has been discouraged due to perceptions of mumps as a self-limited disease and the perceived high cost of mumps vaccines. We estimated the benefits and costs of introducing a mumps vaccination program in Fiji. First, we estimated the burden of mumps and mumps-related complications in Fiji based on the reported cases in the Fiji National Notifiable Disease Surveillance System between 2016 and 2018. We then developed a static simulation model with stable mumps herd immunity after routine measles–mumps–rubella (MMR) vaccination. Finally, we compared the estimated economic burden of mumps with current MR vaccination and the assumptive burden of the stable-state simulation model after routine MMR vaccination. The benefit–cost ratios (BCRs) were 2.65 from the taxpayer view and 3.00 from the societal view. A probabilistic sensitivity analysis indicated that the 1st and 99th percentiles of BCRs were 1.4 and 5.2 from the taxpayer’s perspective and 1.5 and 6.1 from the societal perspective. From both the taxpayer and societal perspectives, the probability of BCRs greater than 1.0 was 100%. A routine MMR program has value for money from both the taxpayer and societal perspectives. MMR vaccination should be urgently introduced in Fiji.

## 1. Introduction

Mumps is a viral infection in humans that primarily affects the salivary glands. Although the majority of mumps cases can be cured naturally without receiving specific treatment, some cases have severe complications such as aseptic meningitis, encephalitis, and hearing loss [1,2]. As of December 2018, the mumps vaccine is on the routine immunization schedule in 122 countries [3]. Live attenuated measles–mumps–rubella (MMR) immunization has dramatically reduced the incidence of mumps [4,5,6,7,8,9]. By contrast, mumps outbreaks have been reported every 3 to 5 years, with case counts ranging from 100 to 1000 per 100,000 population, in countries without regular mumps vaccination programs [4]. Fiji, a country with no routine mumps vaccination program, has seen cyclic mumps outbreaks in recent years. Specifically, from October 2016 through September 2017, more than 200 cases of mumps were reported to the Fiji National Notifiable Disease Surveillance System (NNDSS) on a monthly basis [10,11]. The mumps outbreak peaked in April 2017 with 1462 cases [11]. Although mumps remains endemic in Fiji, the mumps immunization initiative has struggled to gain traction. The lack of willingness of stakeholders to incorporate mumps vaccination into the current measles–rubella (MR) immunization program (i.e., to adopt an MMR vaccination program) in Fiji could be due to their attitudes about the disease; specifically, they regard mumps as a minor, self-limiting illness that does not pose a public health threat [4]. They also believe that the mumps immunization program is expensive compared with current measles–rubella (MR) vaccination [1]. However, recent reports from the Colonial War Memorial (CWM) Hospital have indicated that the incidence of mumps–related hearing impairment and other complications was much higher than what had been previously believed [12,13,14]. Therefore, it is necessary to uncover unambiguous evidence of the MMR vaccination program’s value for money in Fiji. We aimed to conduct a benefit and cost analysis to determine whether Fiji needs to switch from its current MR vaccination program to an MMR vaccination program.

## 2. Materials and Methods

**Figure 1:**
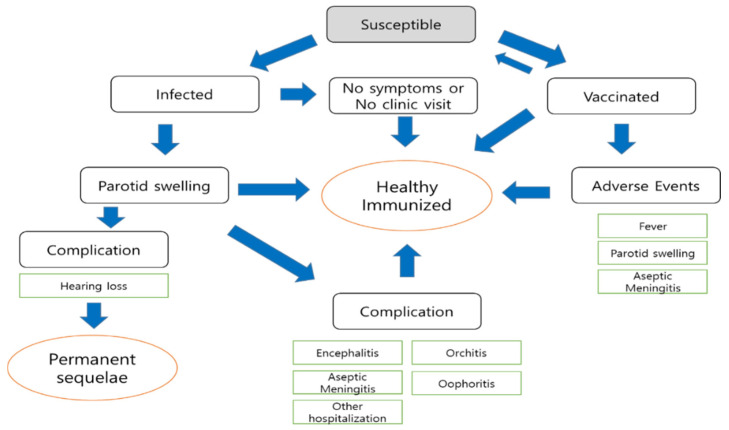
Flowchart of mumps infection and adverse events due to vaccination.

We created a flowchart describing mumps infection and adverse events due to vaccination and used it to estimate the burden of mumps and mumps-related complications in Fiji based on the reported cases in the Fiji NNDSS between 2016 and 2018 (Figure 1). We then developed a static simulation model with stable mumps herd immunity state after routine MMR vaccination. Finally, we compared the estimated economic burden of mumps with current MR vaccination and the assumptive burden of the stable state simulation model after routine MMR vaccination. In the static simulation model, we assumed that Fiji would achieve herd immunity after routine MMR vaccination to compare the current burden with the hypothetically estimated burden. This assumption simplifies the study by eliminating the need to account for population changes or an immunization transition time until Fiji achieves mumps herd immunity. In addition, we assumed that the first dose of the MMR vaccine would be given at the age of 12–18 months and that the age for the second dose would range from 2 years to the age at primary school entry. The benefit–cost ratio was analyzed from the taxpayer view and the societal view. The taxpayer view considers only medical costs, whereas the societal view also considers social costs, especially losses of productivity.

### 2.1. Estimation of Mumps Incidence and Mumps-Related Complications in Fiji

**Figure 2:**
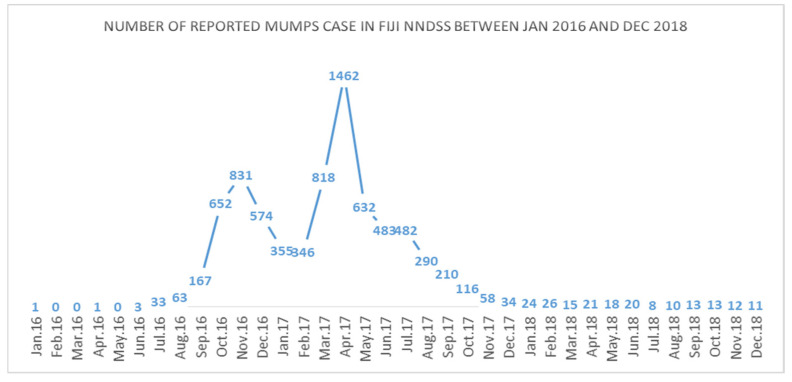
Number of reported mumps case in Fiji NNDSS between Jan. 2016 and Dec. 2018.

The reported cases across Fiji from January 2016 through December 2018 are illustrated in Figure 2. According to the NNDSS in Fiji, a total of 7802 clinical mumps cases were reported in 2016–2018 [11]. This corresponds to an annual rate of 289 new cases of symptomatic mumps per 100,000 population between 2016 and 2018 [11]. However, according to the World Health Organization (WHO) Factsheet, 30% of mumps cases are asymptomatic or have non-specific symptoms [4,15]. Based on this, we assumed that annual mumps incidence in Fiji between January 2016 and December 2018 was 413 cases per 100,000 population. The mumps incidence in Fiji falls within the range of incidence (i.e., 100–1000 new cases per 100,000) that the WHO estimated for countries without an MMR vaccination program [4].

There were no nationwide data for mumps-related complication cases in Fiji between 2016 and 2018. Therefore, we assumed that the incidence of mumps-related complications would be similar to that in other countries with no mumps vaccination. We thus refer to a previous study [16]. According to a large-scale prospective cohort study that enrolled 7502 mumps patients in Japan, the proportion of hearing loss among mumps cases was 0.1% [16]. We believe that the applicability of the results from Japan to Fiji is fairly high based on the fact that there were six reported cases of hearing impairment as a complication of mumps infection in Fiji from January 2016 through December 2018 [12]. Furthermore, the proportion of people with meningitis, encephalitis, and other mumps-related conditions requiring hospitalization in symptomatic mumps cases were estimated at 2.23%, 0.05%, and 1.52% based on a Japanese study [17]. The rates of orchitis among males over 15 and oophoritis among females over 15 caused by mumps were reported to be 38% and 5%, respectively, compared to 0% in those under 15 [18]. The analysis of the complication rate of mumps was restricted to symptomatic patients. Considering the proportion of the mumps incidence in individuals over 15 (4.15%) and that orchitis occurs in only males and oophoritis in only females [18], we estimated that the proportion of orchitis and oophoritis in people caused by mumps infection was 0.79% and 0.10%, respectively. All estimates of the mumps complication rate in symptomatic cases are described in Section 3.

### 2.2. Benefits

#### 2.2.1. Benefits from Prevention of Mumps: Opportunity Cost

The benefits of an MMR vaccination program are the savings in costs that would have been incurred by treating mumps and mumps-related complications if the disease had not been prevented. This opportunity cost is divided into medical and social costs. Medical costs are considered to include all costs for mumps and its complications, including diagnosis, treatment, and rehabilitation. As a social cost, we included the lost productivity of patients or caregivers owing to mumps and its complications. Table 1 shows the medical and social costs incurred by mumps infection and its complications.

#### 2.2.2. MMR Vaccine Effectiveness and Vaccination Coverage

According to the study by Galazaka and colleagues, countries that adopted a two-dose routine mumps vaccination program achieved 97% to 99% reductions in incidence [19]. Among several available mumps vaccine strains, the Leningrad-3 Zagreb vaccine strain has a known protective efficacy ranging from 92-99% in children aged 1–7 years [4]. The MR vaccine coverage was 94% in Fiji [20,21]. We thus assumed that two-dose routine MMR vaccine coverage would be at least 94% since the MMR vaccination program would be incorporated into the existing MR vaccination program. With the 92–99% efficacy of the Leningrad–Zagreb strain vaccine and vaccination coverage of 94%, we assume that 89% of new cases of mumps would be avoidable in Fiji.

#### 2.2.3. Saving Medical Costs

Mumps without complications is a self-limiting disease, but many patients visit outpatient clinics to examine whether mumps complications have occurred. We estimated the costs of parotitis case management without complications based on two outpatient visits: a first visit for mumps diagnosis and a second visit to check for the presence of complications. Patients with symptomatic aseptic meningitis need a single outpatient visit and 10 days of hospitalization including a cerebrospinal fluid (CSF) exam, an X-ray exam, and medication such as Tamiflu. Patients with encephalitis need 1 outpatient visit and 21 days of hospitalization including a CSF examination and medication such as antibiotics. In cases of orchitis and oophoritis, patients need 2 outpatient visits including 1 laboratory examination (including, if applicable, a semen analysis), 1 X-ray examination, and an ultrasound scan. Other mumps complications, for example, pancreatitis, myocarditis, and severe mumps, were assumed to require 5 days of hospitalization. According to a health service costing study in Fiji in 2012, the average unit prices of outpatient clinic visits, admission, laboratory tests, and X-rays at three health facilities were FJD 40.00, FJD 103.93, FJD 19.55, and FJD 16.00, respectively [22]. Transforming the prices of 2012 to those of 2018 with the inflation rate in Fiji between 2012 and 2017 yielded results of FJD 46.57, FJD 120.00, FJD 22.77, and FJD 18.64, respectively [23]. Finally, for each case of parotitis, aseptic meningitis, encephalitis, orchitis, oophoritis, and other mumps-related hospitalizations, taxpayers spend FJD 93.14, FJD 1297.95, FJD 2674.46, FJD 134.55, FJD 134.55, and FJD 692.96, respectively (Table 1).

Table 2 describes the costs of long-term complications. Patients with hearing impairment as a long-term complication of mumps require outpatient clinic visits, hearing aids every 2 years, and hearing tests every year for their remaining life. The average age of the six patients with hearing impairment due to mumps in Fiji between 2016 and 2018 was 16.2 years [12], and the average life expectancy in Fiji was 67.3 years old in 2018 according to the World Bank [24]. Therefore, the remaining lifespan was estimated to be 51.1 years. We assumed that hearing impairment has medical costs of FJD 371.52 per year and FJD 9938.91 for the remaining lifespan, as well as social costs of FJD 85.76 per year and FJD 1147.12 for the remaining lifespan, considering 50% of the minimum wage for the opportunity cost of the time savings.

#### 2.2.4. Saving Social Costs

Productivity loss is calculated as the result of a caregiver’s or patient’s absence from paid labor. We assumed that every sick child needs a full-time caregiver. A child who suffers mumps without complications is unable to attend school for at least 5 days due to the risk of communicability [25]. According to Japanese data, aseptic meningitis, encephalitis, orchitis, oophoritis, and other mumps hospitalizations resulted in productivity losses of 10 days, 22.7 days, 4.9 days, 5.3 days, and 5.0 days, respectively [26]. We assumed the same number of days of productivity loss in Fiji. Patients with hearing impairment due to mumps were assumed to have 4 days of productivity loss for ENT clinic visits and hearing tests every year for their entire lifespan [12]. The minimum wage in Fiji in 2018 was FJD 2.68/h [27]. We thus assumed that a 1-day productivity loss would be FJD 21.44.

We calculated the base case taking 50% of the minimum wage as the opportunity cost of the time savings. We also adjusted the percentage of the opportunity cost of time spent taking care of a sick child to range from 25% to 75% in the sensitivity analysis.

### 2.3. Cost of MMR Vaccination

Additional administrative costs for the MMR vaccine delivery and management would be negligible because the MMR vaccine program could use existing infrastructure, human resources, and vaccination campaigns for the current MR vaccination program. The additional costs that need to be considered for mumps vaccination are the increased cost of the new vaccine and the costs caused by adverse events of the mumps vaccination, if any.

#### 2.3.1. Cost of the Mumps Vaccine

The Fijian government is using the MR vaccine in a 10-dose preparation made by the Serum Institute of India Pvt. Ltd. The price of the vaccine is USD 0.66 per 1-dose preparation in 2019 provided by UNICEF. UNICEF also provides the MMR vaccine in a 1-dose preparation made by the Serum Institute of India Pvt. Ltd with a price of USD 1.28 in 2019 [28]. If the Fijian government purchases the same brand of MMR vaccine instead of the MR vaccine through UNICEF, the Fijian government needs to spend an additional USD 0.63 per 1-dose preparation to include the mumps vaccine. The 10-dose preparation of the MMR vaccine is not expected to be used for 10 children. Dose wastage was also considered. We gave weights to wastage in the rural and urban populations. From two reports on wastage from Iran and Nigeria, we used the worst case to make a conservative estimate [29,30]. Referring to the reported percentage of nationwide wastage in Iran (28% in urban areas and 22% in rural areas), we estimated the weighted mean wastage in Fiji was 25%, considering the distribution of the Fijian population (57% urban and 43% rural) [31]. We thus assumed that the Fijian government would spend an additional USD 0.79 (FJD 1.67: USD 1.00 = FJD 2.00) for one dose of MMR vaccination instead of MR vaccination.

#### 2.3.2. Cost of Mumps Vaccination Adverse Events

MMR vaccine could cause mild adverse events such as fever, pain, rash, and parotid swelling, as well as severe adverse events such as encephalomyelitis, thrombocytopenia, anaphylaxis, and aseptic meningitis [32,33]. It is known that almost all adverse events of the MMR vaccine came from the measles vaccine [33]. According to a study in Japan, the rates of each adverse event from the mumps vaccine were as follows: fever 6.0%, parotid gland swelling 1.8%, and meningitis 0.016% [26]. According to the WHO, mumps vaccines can produce orchitis, oophoritis, pancreatitis, sensory neural hearing loss, and aseptic meningitis [32]. Very few cases of all possible complications have been described in case reports, except for aseptic meningitis, which has a rate of 13–900 cases/10^6^ injections, when using the Leningrad–Zagreb strain [32]. We did not include very rare mumps vaccine adverse events (e.g., encephalitis with a rate of 0.0004% and hearing loss with a rate of 0.000017%) [26]. We assumed that the rates of parotid swelling and aseptic meningitis would be 1.8% and 456.5 cases per 1,000,000 doses, respectively. The severity and cost of parotid swelling and aseptic meningitis due to vaccination were considered the same as those of natural infections [34].

#### 2.3.3. Discounting

We applied a 3% discount rate to both the costs and the health effects. The time horizon was limited to 1 year (2019). We used a static model instead of a dynamic model, inferring what would have happened if Fiji had adopted a routine MMR vaccination program and the coverage had reached beyond the threshold required for herd immunity. We simulated this static model based on the existing literature with similar aims (i.e., exploring the costs and benefits of extending routine immunization to cover mumps vaccination) [26]. The costs incurred and benefits beyond 1 year (e.g., hearing impairment as a long-term complication) were discounted at a 3% rate.

### 2.4. One-Way and Probabilistic Sensitivity Analysis

We calculated the benefit–cost ratio using one-way sensitivity analysis with five parameters as follows: additional vaccine cost, the two-dose MMR vaccine coverage rate, vaccine effectiveness, burden of disease, and the incidence of adverse events [4,20]. We performed a probabilistic sensitivity analysis with 100,000 random draws of the simulation using five parameters: additional vaccine cost, the two-dose MMR vaccine coverage rate, vaccine effectiveness, burden of disease, and the incidence of parotid swelling and incidence of aseptic meningitis after MMR vaccination.

We generated a stochastic model with the following parameters: vaccine effectiveness (beta distribution, alpha = 95, beta = 5); vaccine coverage (beta distribution, alpha = 31.02, beta = 1.98); burden of disease (log-normal distribution, mean = log(3715), SD = 0.24321); vaccine cost (log-normal, mean = log(1.67), SD = 0.11); incidence rate of parotid swelling after vaccination (beta distribution, alpha = 8.8, beta = 541.2); incidence rate of aseptic meningitis after vaccination (beta distribution, alpha = 9.074, beta = 19986.38); and productivity loss rate (beta distribution, alpha = 7, beta = 7). We pulled samples from each distribution independently, and thus there was no correlation between variables.

Low and high parameter values, and their distributions, were described in the Supplementary Material (Table S1). We used R software (v4.1.2, R Core Team 2021) and python (3.9) for analysis. Python syntax for the sensitivity analysis are described in the Supplementary Material (Text S1).

## 3. Results

### 3.1. Health Effects from the MMR Vaccination

Between 2016 and 2018, 6974 cases of mumps, 155 cases of meningitis, 3 cases of encephalitis, 55 cases of orchitis, 7 cases of oophoritis, 106 cases of mumps-related hospitalization, and 7 cases of hearing impairment would have been avoided if the MMR vaccine had been introduced instead of the MR vaccine in Fiji’s national immunization program.

### 3.2. Benefits

The estimated benefits from preventing mumps, mumps complications, and the burdens of each complication are presented in Table 3. Based on the 89% reduction in patients and complications of mumps, the annual benefits from saving medical costs and social costs were estimated at FJD 337,307.94 and FJD 136,110.17, respectively.

### 3.3. Costs

#### 3.3.1. Annual Vaccine Costs

According to the Fiji population and housing census in 2017, the population of 0- to-4 year-olds and 5- to 9-year-olds was 91,897 and 88,295, respectively [31]. Considering a two-dose MMR vaccination program at 12 months old and before primary school entry, the annual number of MMR vaccination candidates for the first and second doses are approximately 18,379 and 17,659, respectively. The total annual number of candidates is 36,038. Considering a 94% vaccine coverage rate, we assumed that 33,876 doses of MMR vaccines, costing an additional FJD 56,572 per year, would be necessary for all candidates.

#### 3.3.2. Costs from Increased Adverse Events of MMR Vaccination

The estimated incidence and burden of each additional adverse event of MMR vaccination are calculated in Table 4. The additional annual medical costs and social costs lost due to MMR vaccination adverse events were FJD 70,575.30 and FJD 30,711.40, respectively.

### 3.4. Benefit–Cost Ratio of Implementing a Two-Dose MMR Vaccination Program 

From the taxpayer’s perspective, the medical benefit from decreasing the medical burden of mumps was FJD 337,307.94, and the medical and vaccine costs of MMR vaccination were FJD 127,148.22. Therefore, the benefit–cost ratio was 2.65. From the societal perspective, considering not only medical costs, but also social costs, the benefit–cost ratio was 3.00, with a FJD 473,418.11 total benefit and a FJD 157,859.62 total cost if a stable state of MMR vaccination is achieved. All benefits, costs and benefit-cost ratios are calculated in Table 5.

### 3.5. Sensitivity Analysis

#### 3.5.1. One-Way Sensitivity Analysis

**Figure 3:**
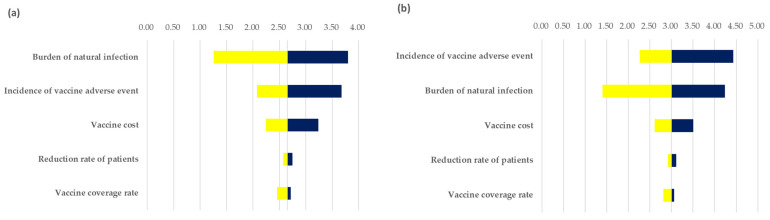
One-way analysis results (tornado plot of the benefit–cost ratio): (**a**) taxpayer’s perspective; (**b**) societal perspective.

In the one-way sensitivity analysis, five parameters had reasonable variation effects on the benefit–cost ratios, but all ratios were larger than 1.0 from the taxpayer and social perspectives. Figure 3 shows that the burden of mumps infection and incidence of mumps vaccination adverse events were variables that influenced the benefit–cost ratio.

#### 3.5.2. Probabilistic Sensitivity Analysis

**Figure 4:**
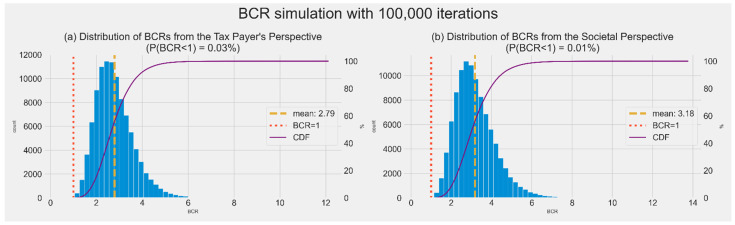
Cumulative distribution function(CDF) of benefit–cost ratios (BCRs) from the societal perspective and taxpayer’s perspective with 100,000 iterations of random sampling of five parameters.

Figure 4 presents the distribution of the benefit–cost ratio with 100,000 iterations of random sampling of seven parameters. A probabilistic sensitivity analysis indicated that the 1st and 99th percentiles of BCRs were 1.4 and 5.2 from the taxpayer’s perspective and 1.5 and 6.1 from the societal perspective. From both the taxpayer and societal perspectives, the probability of BCRs greater than 1.0 was 100%.

## 4. Discussion

Our research shows that a two-dose regular MMR immunization program would be worthwhile in Fiji in terms of the benefit–cost ratio. In every situation, the benefit outweighed the cost. Considerable benefits were also suggested in many other countries such as Austria (3.6), the USA (7.4), Israel (5.9) and Japan (5.9) [17,18,35]. If the Fijian government adopts a routine MMR immunization program, it would have financially positive results. Meanwhile, the annual budget incrementally incurred for running the MMR program (56,572 FJD/year) is affordable by the Fijian government, as this program would account for 0.017% of annual budget of the Ministry of Health, Fiji, and this sum is less than the contingency budget [36]. The largest challenge in expanding the routine immunization services in Fiji has been a lack of evidence showing the economic benefits of MMR vaccination. This study suggests that including mumps in the current vaccination program would have profound value in terms of money [37].

The WHO recommends routine mumps vaccination programs in countries with a well-established childhood vaccination program maintaining more than 80% coverage of MR vaccination [4]. Fiji meets all these criteria [20]. According to the WHO, countries without regular mumps vaccination programs experience epidemics at intervals of 2–5 years [4]. This would indicate that Fiji might possibly have the next mumps outbreak between 2020 and 2022. Acquired sensorineural deafness caused by mumps is one of the major causes of childhood deafness, including in Fiji [4,12]. If the MMR vaccination program is implemented in Fiji, six or seven children will be protected from hearing loss every 3 years. This is why the introduction of mumps immunization in Fiji is critical.

During the Sixth Heads of Health Meetings in 2018, policymakers from Pacific Island countries, including Fiji, agreed that developing a comprehensive immunization schedule for the region should be a top priority. Mumps immunizations are included in this program [37]. Still, mumps vaccination programs have not been implemented in Fiji, Solomon Islands, Tonga, Tuvalu, Kiribati, Vanuatu, and Nauru [37].

The key lessons learned from countries with similar economic situations that have included mumps vaccination in the routine program are as follows: (1) intensive collaboration between, and concerted efforts by, governments and United Nations agencies, particularly, the United Nations’ Children’s Fund, are important; (2) strong political will for incorporating mumps into the current vaccination program is critical; and (3) awareness raising for MMR vaccination is a prerequisite [4]. In addition, we could take lessons from other countries with different economic situations. In the 1980s, Denmark and the Netherlands resisted the introduction of the mumps vaccine for political reasons and concerns about side effects, although other developed countries had already started mumps vaccination programs. However, they decided to introduce the vaccine when they obtained evidence for the safety of the vaccine and its economic benefits [38]. Mumps cases in Slovenia and Croatia dramatically decreased after the introduction of routine mumps vaccination (410 cases/10^6^ in 1979 to 4 cases/10^6^ in 1995 and from 101 cases/10^6^ in 1985 to 12 cases/10^6^ 1995, respectively) [1]. It is also worth noting that the WHO recommends surveillance and monitoring for any possible side effects after implementing an MMR vaccination program [4].

If the Fijian government decides to introduce a mumps vaccine, the two-dose MMR vaccine program is strongly recommended because of its disease-protective effectiveness and cost savings [17]. As the majority of children acquire mumps infections before the age of 10 years, the first dose of the MMR vaccine should be given at the age of 12–18 months, and the age for the second dose ranges from 2 years of age to the age at primary school entry with a minimum of a 1-month interval [4]. This protocol would target the age group most susceptible to mumps. When planning to use a mumps vaccine, a country should plan to provide guidelines for monitoring, investigating, and managing adverse events and training health workers about how to communicate risk and provide health education [4]. Immunization plays an essential role in achieving the sustainable development goals and is a key priority among other health and social services [39].

There are several limitations of this study. First, we carried out static modeling estimation with an assumption that Fiji would have already reached herd immunity through the mumps vaccine. The reason for doing so was to make the analysis simple with no need to consider population changes and the immunization transition period before achieving mumps herd immunity in Fiji. Therefore, the study results only mean that Fiji could receive more benefits than costs from MMR vaccination if it achieves mumps herd immunity. Second, the mumps disease burden in Fiji substantially varies year by year depending on the existence of outbreaks. We assume that mumps outbreaks take place in Fiji every 3 years based on the WHO reports indicating that mumps outbreaks occur every 2–5 years if there are no regular mumps vaccination programs [4]. Third, we estimated the mumps complication rate and hospitalization dates using Japanese research. These parameters might be different in the context of Fiji. Similarly, the incidence of mumps complications in Fiji may be different from other countries, but we used the incidence rate of other countries because no data were available for Fiji. Fourth, we neglected mortality and permanent sequelae due to mumps encephalitis (e.g., paralysis or seizure) because the incidence was very low. Accounting for these complications would have increased the benefit–cost ratio.

## 5. Conclusions

A routine two-dose MMR program instead of the current MR vaccination program in Fiji is cost-saving from both the taxpayer and societal perspectives. A routine two-dose MMR vaccination program should be introduced in Fiji as soon as possible to prevent regular mumps outbreaks. Expanding the immunization program to include MMR vaccination is the foundation of universal health coverage and primary health care systems.

## Figures and Tables

**Table 1:** Medical and social costs of mumps and short-term complications of mumps in Fiji.

Mumps Short-Term Complica-Tions	Medical Costs					Social Costs	
	Outpatient Visit (Time)	Admission (Days)	Lab Test (Time)	X-ray (Time)	Unit Costs (FJD)	Productivity Loss (Days)	Unit Costs (FJD)
	FJD 46.57 /Time	FJD 121.00 /Day	FJD 22.77 /Time	FJD 18.64 /Time		FJD 21.44 /Day	
Mumps (parotitis)	2	0	0	0	93.14	5	53.60
Aseptic meningitis	1	10	1	1	1297.95	10	107.20
Encephalitis	1	21	3	1	2674.46	22.7	243.34
Orchitis	2	0	1	1	134.55	4.9	52.53
Oophoritis	2	0	1	1	134.55	5.3	56.82
Other mumps-related hospitalizations	1	5	1	1	692.96	5	53.60

**Table 2:** Medical and social costs of long-term mumps complications in Fiji.

Mumps Long-Term Complica-Tions	Medical Cost					Social Cost		
	Outpatient Visit (Time)	Hearing Aid (Year)	Lab Test (Time)	Unit Costs (FJD)/Year	Unit Costs (FJD) for Life	Productivity Loss (Days)	Unit Costs (FJD)/Year	Unit Costs (FJD) for Life
	FJD 46.57 /Time	FJD 232.84 /Year	FJD 22.77 /Time		51.1 Year 3% Discount/Year	FJD 21.44 /Day		51.1 Year 3% Discount/Year
Permanent hearing impairment	2	1	2	371.52	9938.91	4	85.76	1147.12

**Table 3:** Estimated benefits from preventing mumps, mumps complications, and the burdens of each complication.

Mumps Complications	Incidence (%)	Number of Cases	Reduction in the Number of Cases	Medical Costs per Case (Fj $)	Social Costs per Case (Fj $)	Saved Medical Costs (Fj $)	Saved Social Costs (Fj $)
Parotid swelling	100.00	2600.50	2324.85	93.14	53.60	216,529.92	12,461.80
Aseptic meningitis	2.23	57.99	51.84	1297.95	107.20	67,291.01	5557.69
Encephalitis	0.05	1.30	1.16	2674.46	243.34	3108.86	282.87
Orchitis	0.79	20.54	18.37	134.55	52.53	2471.16	964.74
Oophoritis	0.10	2.60	2.32	134.55	56.82	312.80	132.09
Hearing loss	0.10	2.60	2.32	9938.91	1147.12	23,106.44	2666.88
Other hospitalizations	1.52	39.53	35.34	692.96	53.60	24,487.76	1894.10
Total						337,307.94	136,110.17

**Table 4:** The estimated incidence and burden of additional adverse events of MMR vaccination.

Adverse Effect of Mumps Vaccine	Incidence (%)	Number of Cases	Medical Costs per Case (FJD)	Social Costs per Case (FJD)	Total Medical Costs (FJD)	Total Social Costs (FJD)
Parotid swelling	1.60	542.01	93.14	53.6	50,481.48	29,051.82
Aseptic meningitis	0.0457	15.48	1297.95	107.2	20,093.82	1659.59
Total					70,575.30	30,711.40

**Table 5:** The benefit–cost ratio of MMR vaccination.

Benefits		Costs			Taxpayer’s Perspective		Societal Perspective	
Medical Benefits (1)	Social Benefits (2)	Medical Costs (3)	Social Costs (4)	Vaccine Costs (5)	Net Present Value (1) − (3) − (5)	Benefit–Cost Ratio (1)/((3) + (5))	Net Present Value (1) + (2) − (3) − (4) − (5)	Benefit–Cost Ratio ((1) + (2))/((3) + (4) + (5))
337,307.94	136,110.17	70,575.30	30,711.40	56,572.92	210,159.72	2.65	315,558.49	3.00

## Data Availability

Data can be shared upon request under the control of Ministry of Health, Fiji (contact eric.rafai@govnet.gov.fj).

## Supplementary Materials

The following are available online at https://www.mdpi.com/article/10.3390/ijerph19031861/s1, Table S1: Parameter values and justification for the sensitivity analysis; Text S1. Syntax for the sensitivity analysis. File S1. Mumps cases report raw data; File S2. Mumps related hearing impairment in CWM hospital between 2016–2018; File S3. Distribution parameters.
