# Supplementary material for: An Economic Analysis of Mumps Vaccination in Fiji: Static Model Simulation of Routine Measles–Mumps–Rubella (MMR) Vaccination Instead of Current Measles–Rubella (MR) Vaccination
Source: Int J Environ Res Public Health. 2022 Feb 7;19(3):1861. doi: 10.3390/ijerph19031861 (PMC8835634; doi:10.3390/ijerph19031861)
Supplement: Supplementary file 1 [file ijerph-19-01861-s001.zip › File S2. Mumps realated hearing impairment in CWM hospital between 2016-2018.pdf]

File S2. Mumps related hearing impairment at ENT clinic, CWM hospital, Jan 2016-Dec 2018

Age/	Sex/ Ethnicity	Salivary gland swelling	Recognition hearing impairment	Affected side	ENT clinic visit	Ear drum	Diagnosis	Hearing Grade
12	Female/ Indo-Fijian	Aug, 2017	After 5 days swelling	Left	Dec, 2017	Normal	Pure Tone Audiometer	Severe SNHL
14	Male/ Itoke	Dec, 2016	After swelling, unknown	Right	Dec, 2018	Normal	Pure Tone Audiometer	Severe SNHL
13	Female/ Itoke	May, 2017	After swelling, unknown	Left	May, 2018	Normal	Pure Tone Audiometer	Moderate SNHL
13	Female/ Indo-Fijian	Mar, 2017	After 2 weeks swelling	Right	Mar, 2019	Normal	Voice test Tuning Fork	Severe SNHL
13	Male/ Indo-Fijian	Dec, 2018	After 3 days swelling	Left	Aug, 2019	Normal	Pure tone audiometer	Profound SNHL
32	Female/ Indo-Fijian	June, 2017	After 5 days swelling	Right	Nov, 2017	Normal	Pure tone audiometer	Profound SNHL
